# Comparative Antibacterial Activity of Cabbage Varieties Against Thermophilic *Bacillus *spp. Isolated from Wheat Grains

**DOI:** 10.3390/foods15030600

**Published:** 2026-02-06

**Authors:** Liliya Alashbayeva, Madina Yakiyayeva

**Affiliations:** 1 Faculty of Food Technology, Dulaty University, Taraz 080000, Kazakhstan; alashbaeval@gmail.com; 2 Research Institute of Food Technologies, Almaty Technological University, Almaty 050012, Kazakhstan

**Keywords:** wheat flour, *Bacillus subtilis* and *Bacillus mesentericus*, cabbage varieties, antimicrobial activity, plant juices

## Abstract

The microbiological safety of whole wheat flour remains a critical issue due to its susceptibility to contamination by spore-forming thermophilic bacteria. In this study, two thermophilic species, Bacillus subtilis and Bacillus mesentericus, were isolated from locally produced wheat grains and used as target microorganisms to evaluate the antibacterial potential of freshly pressed cabbage juices. Juices obtained from five cabbage varieties—red cabbage, white cabbage, napa (Chinese) cabbage, broccoli, and cauliflower—were comparatively assessed using the broth dilution method to determine their minimum inhibitory and bactericidal effects (*n* = 3). The results revealed pronounced differences in antibacterial efficacy among the tested samples. White cabbage juice exhibited selective inhibitory activity against *B. subtilis* at a dilution of 1:4, whereas napa cabbage and broccoli juices demonstrated the highest antibacterial activity against both *Bacillus* species at a dilution of 1:3. Importantly, napa cabbage juice showed no inhibitory effect on *Saccharomyces cerevisiae*, indicating its compatibility with dough fermentation processes. Spectroscopic analysis of the bioactive fraction obtained from napa cabbage juice revealed characteristic absorption bands at 3422 cm^−1^ (O–H stretching), 2907–2840 cm^−1^ (aliphatic C–H stretching), 1740 cm^−1^ (ester carbonyl group), and 1641 cm^−1^ (C=C stretching). The predominance of lipophilic compounds, including fatty acid esters, terpenes, and sulfur-containing compounds (734 cm^−1^), suggests a molecular basis for the observed antibacterial activity against *Bacillus* spp. Overall, these findings identify napa cabbage as a promising source of selective natural antimicrobial agents capable of enhancing the microbiological safety of whole wheat flour-based bakery products without compromising yeast activity.

## 1. Introduction

In recent years, increasing attention has been directed toward the development of bakery products based on whole-grain flour due to their enhanced nutritional value and relevance to healthy diets [1,2,3,4,5]. Whole-grain products retain the bran and germ fractions, which are rich in dietary fiber, vitamins, minerals, and bioactive compounds, including phenolics and antioxidants [1,2,3,4,6,7]. However, the preservation of the outer grain layers also increases the susceptibility of whole wheat flour to microbiological contamination, posing challenges for product quality, shelf life, and safety [8,9,10,11,12].

Among the microorganisms associated with cereal-based products, spore-forming thermophilic bacteria of the genus *Bacillus* are of particular concern [11,13]. Species such as *Bacillus subtilis* and *Bacillus mesentericus* are commonly detected in wheat grains and flour and are capable of surviving adverse processing conditions due to their ability to form heat-resistant endospores [14,15]. During bread baking, although oven temperatures may reach approximately 200 °C, the internal crumb temperature typically remains within 80–90 °C, which is insufficient for complete spore inactivation [16,17,18]. Consequently, viable spores may persist and contribute to microbial spoilage and quality deterioration during storage [19,20,21].

Conventional strategies to control microbial growth in bakery products often rely on synthetic preservatives; however, increasing consumer demand for natural and minimally processed foods has stimulated interest in biologically based alternatives [22,23]. In this context, plant-derived antimicrobial compounds have attracted significant attention as potential natural agents capable of improving food safety while maintaining product quality and consumer acceptance [18,22,24,25,26].

Vegetables belonging to the genus *Brassica*, particularly cabbage species, are known to be rich in biologically active compounds such as glucosinolates and their degradation products (isothiocyanates), organic acids, polyphenols, anthocyanins, and sulfur-containing compounds [1,2,3,10,11,12,13,14,27,28]. Numerous studies have demonstrated that extracts and juices obtained from *Brassica oleracea* varieties exhibit antioxidant, antimicrobial, and antifungal activities against a wide range of food-associated microorganisms [29,30,31,32,33,34]. Freshly pressed cabbage juice represents a minimally processed matrix that preserves water-soluble and heat-sensitive bioactive constituents and has been reported to exhibit antimicrobial activity without the use of organic solvents or aggressive extraction conditions [18,22,35,36,37].

Due to its natural origin and technological compatibility, cabbage juice is of particular interest for applications in bakery systems, where interference with yeast-driven fermentation must be avoided [38,39,40,41]. Selective antimicrobial activity against spoilage and spore-forming bacteria, combined with the absence of inhibitory effects on *Saccharomyces cerevisiae*, represents a key requirement for such applications [22,35,41,42].

The aim of the present study was to evaluate the antibacterial activity of cabbage-derived juices and extracts against thermophilic *Bacillus* spp. isolated from wheat grains and to assess their compatibility with yeast strains relevant to dough fermentation. In addition, spectroscopic characterization of selected bioactive extracts was performed to provide insight into the chemical groups and compounds potentially responsible for the observed antimicrobial effects [14,22,43,44,45].

## 2. Materials and Methods

### 2.1. Microbiological Control of Bread

To identify thermophilic bacteria present in wheat grains, which are the main raw material used in bread production, a classical heat treatment method was employed using wheat grain-derived microorganisms as test strains, as described in previous studies on spore-forming bacteria isolation [46,47]. In this procedure, 100 g of wheat grains were placed into a sterile container, and 1 L of distilled water was added. The resulting suspension was heated at 70–80 °C for 30 min, a treatment designed to eliminate vegetative cells while selectively preserving heat-resistant spore-forming bacteria [48,49].

Following heat treatment, the samples were incubated in a thermostat at 25–30 °C for 2–3 days. During the incubation period, the growth and development of thermophilic and spore-forming microorganisms were monitored. This approach enabled the selective isolation of thermophilic *Bacillus* spp. naturally present in wheat grains and provided suitable cultures for subsequent microbiological characterization [50,51].

Primary identification of the isolates, purification of cultures, and characterization of morphological, staining, and physiological–biochemical properties were performed according to standard microbiological procedures described in Bergey’s Manual of Systematic Bacteriology [52,53].

The quantitative determination of spore-forming bacteria was carried out using the Koch plate count method, which is widely applied in food microbiology [53,54]. Serial dilutions of the samples were prepared, and aliquots from each dilution were inoculated onto solid tryptic soy agar (TSA). The inoculated plates were incubated in a thermostatic chamber (electric dry-air thermostat TC-1/80 SPU, Smolensk SKTB SPU, Russia) at 37 ± 1 °C for 72 h.

A series of biochemical, staining, and morphological tests was conducted to characterize the isolates in accordance with established protocols [52,55]:

− Motility was assessed by stab inoculation into semi-solid agar;
− Lecithinase activity was determined using egg yolk–salt agar (EYSA);
− Catalase activity was evaluated using 3% hydrogen peroxide;
− Starch hydrolysis and fermentation of mannitol, salicin, and xylose were examined using carbohydrate-containing media with phenol red as a pH indicator;
− Hemolytic activity was assessed on 5% blood agar plates;
− The Voges–Proskauer reaction was performed using standardized MR–VP medium.

The staining properties of the isolates were examined by Gram staining, followed by microscopic observation using an inverted microscope (A. KRÜSS Optronic MBL3200, Hamburg, Germany), as recommended for preliminary identification of *Bacillus* spp. [52].

#### Extraction Procedures

Plants belonging to the cabbage group were used as sources of antimicrobial compounds, including red cabbage (*Brassica oleracea* var. *capitata* f. *rubra*), white cabbage (*Brassica oleracea* var. *capitata*), napa (Chinese) cabbage (*Brassica rapa* subsp. *pekinensis*), broccoli (*Brassica oleracea* var. *italica*), and cauliflower (*Brassica oleracea* var. *botrytis*). All plant materials were purchased from a local market in Taraz, Zhambyl region, Republic of Kazakhstan, in 2025. Prior to microbiological analysis, the plant materials were processed using a juice extractor and subsequently sterilized by filtration through syringe filters with a pore size of 0.22 μm.

**Figure 1:**
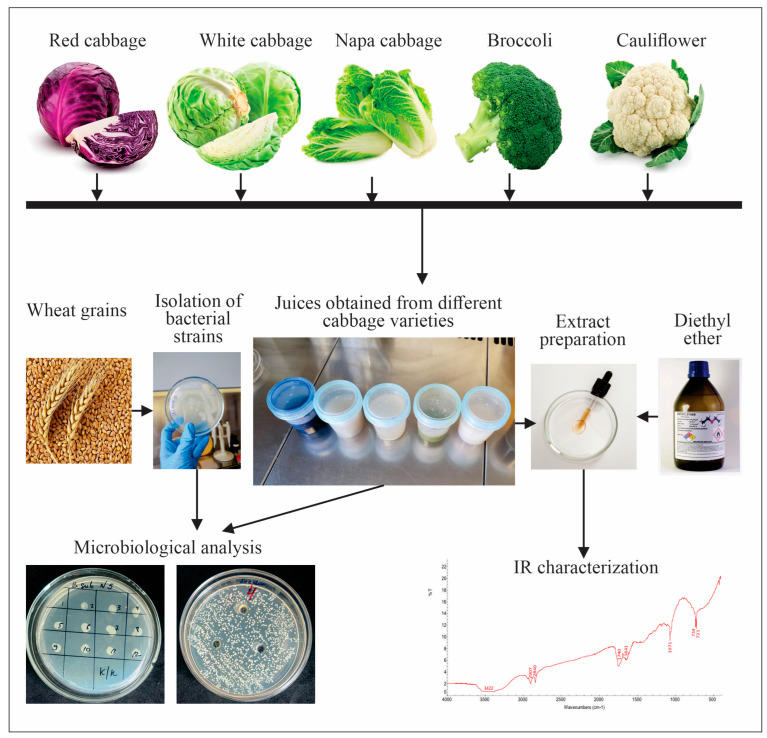
Schematic overview of the experimental procedures used in the study of cabbage varieties.

The overall experimental workflow and the stages of sample preparation are presented in Figure 1. This schematic illustrates the sequence of operations applied during extract preparation, including raw material processing, solvent application, and recovery of the obtained extracts.

All cabbage samples were subjected to an identical extraction protocol. Approximately 1.5 kg of each cabbage type was chopped into small pieces and placed into a 5 L glass container, after which 1.5 L of analytical-grade diethyl ether was added. The containers were tightly sealed to prevent solvent evaporation. To ensure efficient extraction of lipophilic constituents, the mixtures were kept at room temperature for more than 7 days.

After completion of the extraction period, the vessels were opened and the liquid phase was carefully decanted into a large beaker. During extraction, two distinct phases were formed, which were subsequently separated using a separatory funnel. The upper, light-yellow organic phase contained lipophilic cabbage constituents dissolved in diethyl ether, whereas the lower, more viscous orange-colored phase represented a denser fraction.

The upper organic phase was concentrated under reduced pressure at 40 °C using a rotary evaporator (Hei-VAP Core, Schwabach, Germany), yielding approximately 500 mg of dry extract, corresponding to an average extraction yield of 0.06% relative to the initial raw material weight. The obtained extract was stored at −28 °C until further chemical characterization and antimicrobial and antifungal evaluation.

The lower dense phase was placed under a fume hood to allow complete evaporation of residual solvent. After solvent removal, this fraction transformed into a highly viscous, dark-orange, honey-like substance, which is characteristic of prolonged organic solvent extraction of plant materials. This fraction was also stored under frozen conditions and subsequently used for additional chemical analyses and biological activity assays.

### 2.2. Evaluation of Antimicrobial Properties

Antimicrobial activity was evaluated using the broth dilution method with two-fold serial dilutions. The procedure was performed in accordance with internationally accepted guidelines, including the CLSI document *Performance Standards for Antimicrobial Susceptibility Testing* (CLSI M100-S25, 2015, Vol. 35, No. 3), as well as the Russian methodological recommendations *Guidelines for Determining the Sensitivity of Microorganisms to Antibacterial Agents* (MUK 4.12.1890-04, Moscow, 2004).

Minimum inhibitory concentration (MIC) and minimum bactericidal concentration (MBC) of the cabbage juices against *Bacillus subtilis* and *Bacillus mesentericus* were determined using a two-fold serial dilution method in Mueller–Hinton broth. The inoculum was standardized to 0.5 McFarland (≈1.5 × 10^8^ CFU/mL) and added to each tube to obtain a final bacterial concentration of ≈1.5 × 10^6^ CFU/mL. Tubes were incubated at 37 ± 1 °C for 18–24 h.

MIC was defined as the lowest juice dilution (highest concentration) showing no visible growth (no turbidity) after incubation. To determine MBC, aliquots from tubes with no visible growth were subcultured onto nutrient agar and incubated at 37 ± 1 °C for an additional 18–24 h. MBC was defined as the lowest juices dilution at which no colonies were recovered on agar plates, indicating complete loss of bacterial viability.

All assays were performed in triplicate. The experimental work was carried out using the following laboratory equipment: DEN-1 densitometer (Biosan, Riga, Latvia); Comfort thermoshaker (Eppendorf, Hamburg, Germany); LB 210-A analytical balance (A&D, Moscow, Russia); PB11 pH meter (Sartorius, Göttingen, Germany); Systec V-120 vertical autoclave (Systec GmbH, Osnabrück, Germany); BD-115 incubator (Binder GmbH, Tuttlingen, Germany); BioIIA/G laminar airflow cabinet (Telstar, Terrassa, Spain); MS3 Digital orbital shaker (IKA, Staufen, Germany); Eppendorf adjustable micropipettes (1–10 mL, 100–1000 µL, 20–200 µL, and 0.5–10 µL; Eppendorf, Hamburg, Germany); Haake P14 thermostatic water bath (Thermo Fisher Scientific, Karlsruhe, Germany); and an Arium 611 VF water purification system (Sartorius, Göttingen, Germany).

#### 2.2.1. Total Phenolic Content (TPC)

The total phenolic content (TPC) of the cabbage extracts was determined using the Folin–Ciocalteu colorimetric method with minor modifications. Briefly, 0.1 mL of the extract was mixed with 0.5 mL of Folin–Ciocalteu reagent previously diluted tenfold with distilled water. After incubation for 5 min at room temperature, 0.4 mL of 7.5% (*w*/*v*) sodium carbonate solution was added. The reaction mixture was then incubated in the dark for 30 min at ambient temperature to allow color development.

Absorbance was measured at 765 nm using a UV–Vis spectrophotometer. Gallic acid was used as the reference standard to construct a calibration curve in the concentration range of 0–200 mg/L. The TPC results were expressed as milligrams of gallic acid equivalents per gram of dry extract (mg GAE/g). All measurements were performed in triplicate (*n* = 3).

#### 2.2.2. Total Flavonoid Content (TFC)

The total flavonoid content (TFC) was quantified using the aluminum chloride (AlCl_3_) colorimetric assay. Briefly, 0.5 mL of the extract was mixed with 0.5 mL of a 2% (*w*/*v*) AlCl_3_ solution prepared in ethanol. The mixture was incubated for 15 min at room temperature in the dark.

Absorbance was recorded at 415 nm using a UV–Vis spectrophotometer. Quercetin was used as the reference compound for the calibration curve over a concentration range of 0–100 mg/L. The results were expressed as milligrams of quercetin equivalents per gram of dry extract (mg QE/g). All analyses were conducted in triplicate (*n* = 3).

### 2.3. FTIR Spectroscopic Analysis

Fourier-transform infrared (FTIR) spectra were recorded using a Nicolet iS50 spectrophotometer (Thermo Fisher Scientific, Waltham, MA, USA). The analysis was conducted in accordance with standard procedures for solid samples, with dried cabbage extracts analyzed in transmission mode. Briefly, 3 mg of each dried extract was thoroughly mixed with 100 mg of spectroscopic-grade potassium bromide (KBr) using an agate mortar and pestle until a homogeneous, finely ground mixture was obtained. The resulting mixture was then compressed into a transparent pellet using a hydraulic press at a pressure of 10 t/cm^2^, forming a disk with a diameter of 1 cm. The prepared pellet was placed into the sample holder for spectral acquisition.

Infrared spectra were recorded over the wavenumber range of 400–4000 cm^−1^ with a spectral resolution of 2 cm^−1^. For each sample, 100 scans were accumulated to improve the signal-to-noise ratio.

### 2.4. Experimental Design and Data Analysis

The experimental design of the study was based on a comparative in vitro evaluation of the antibacterial activity of freshly pressed cabbage juices and selected cabbage-derived extracts against thermophilic *Bacillus* spp. isolated from wheat grains. Two bacterial species, *Bacillus subtilis* and *Bacillus mesentericus*, were used as target microorganisms due to their relevance to microbiological spoilage of whole wheat flour and bakery products.

Five cabbage varieties (red cabbage, white cabbage, napa (Chinese) cabbage, broccoli, and cauliflower) were included in the experimental design. Each juice sample was tested independently against both bacterial strains using a two-fold broth dilution assay to determine minimum inhibitory concentration (MIC) and minimum bactericidal concentration (MBC). All microbiological assays were performed in triplicate (*n* = 3) to ensure reproducibility and reliability of the results.

In parallel, the effects of cabbage juices on *Saccharomyces cerevisiae* were evaluated to assess compatibility with dough fermentation processes. Based on the combined assessment of antibacterial efficacy against *Bacillus* spp. and selectivity toward yeast cells, napa (Chinese) cabbage was selected for further extraction and chemical characterization.

Spectral analysis using FTIR spectroscopy was applied as a qualitative analytical tool to characterize functional groups present in the bioactive fraction of the selected extract. FTIR measurements were conducted in triplicate, and representative spectra were used for interpretation of dominant absorption bands associated with antibacterial activity.

For data analysis, MIC and MBC values were expressed as dilution ratios corresponding to the lowest concentration showing inhibitory or bactericidal effects. Results were summarized using descriptive statistics. Due to the qualitative and screening-oriented nature of the study, no inferential statistical comparisons were applied. Consistency across replicates was verified by concordance of results obtained in independent experimental runs.

### 2.5. Statistical Analysis

All experiments were performed in triplicate (*n* = 3), and the results are presented as mean values ± standard deviation (SD). Minimum inhibitory concentration (MIC) and minimum bactericidal concentration (MBC) values were defined as the lowest dilution at which consistent inhibitory or bactericidal effects were observed in at least two out of three independent replicates. Given the exploratory and screening-based nature of the study, which was focused on the comparative assessment of antibacterial activity among different cabbage varieties rather than hypothesis testing, inferential statistical analyses were not applied. The reliability and reproducibility of the results were ensured by strict standardization of experimental conditions, identical inoculum preparation, and repeated assays performed independently. This approach is commonly applied in preliminary antimicrobial screening studies aimed at identifying promising bioactive sources for further in-depth quantitative and mechanistic investigations.

## 3. Results

### 3.1. Isolation of Bacillus *spp.* from the Investigated Flour Samples

After the incubation period, representatives of the genus *Bacillus* were detected only in the first dilution (1 × 10^−1^). Two distinct colony morphotypes were observed and subsequently subcultured onto tryptic soy agar (TSA) plates for further characterization. Quantitative analysis revealed that the first morphotype, characterized by dry, rough, light-yellow colonies, reached a concentration of 80 CFU/g of dry flour, whereas the second morphotype, presenting as dry, wrinkled colonies with a yellowish-brown coloration, accounted for 20 CFU/g.

Microscopic examination of smears prepared from both colony types revealed Gram-positive, rod-shaped bacteria containing endospores. Cells of the first morphotype were predominantly observed as single rods or arranged in short chains. In contrast, cells of the second morphotype appeared as slender Gram-positive rods, occasionally forming short filamentous structures.

The enzymatic and hemolytic activities of the isolated cultures were further evaluated. Neither morphotype exhibited lecithinase activity, as indicated by the absence of turbidity zones or opalescent halos on egg yolk–salt agar. However, cultivation of the second morphotype on blood agar resulted in the formation of a greenish-brown discoloration surrounding the colonies, indicative of α-hemolytic activity. No visible changes in the medium were observed around colonies of the first morphotype, suggesting the absence of hemolytic properties.

A summary of the morphological, staining, and biochemical characteristics of the isolated strains is presented in Table 1.

Based on the combined evaluation of morphological, cultural, staining, and biochemical characteristics, Colony No. 1 was identified as a representative of Gram-positive, rod-shaped bacteria forming centrally located endospores. On solid nutrient media, the isolate produced dry, rough colonies with a light-yellow pigmentation, which is characteristic of aerobic spore-forming *Bacillus* species. Microscopic examination confirmed the presence of endospores located predominantly in the central region of the vegetative cells, without causing significant cell swelling. The isolate exhibited active motility and pronounced catalase activity, further supporting its affiliation with the genus *Bacillus*.

The strain demonstrated a positive Voges–Proskauer reaction, indicating the ability to ferment glucose via the butanediol fermentation pathway under anaerobic conditions. In addition, the isolate showed the ability to hydrolyze starch, reflecting the presence of extracellular amylolytic enzymes. At the same time, no fermentation of xylose, salicin, or mannitol was detected, which is consistent with the biochemical profile of *Bacillus subtilis* described in standard taxonomic keys. Taken together, the observed phenotypic and biochemical traits closely correspond to those typically reported for *Bacillus subtilis*, allowing this isolate to be reliably identified at the species level.

Colony No. 2 exhibited distinct morphological and biochemical features. On solid nutrient medium, the isolate formed dry, wrinkled colonies with a yellowish-brown coloration, clearly differentiating it from Colony No. 1. Microscopic analysis revealed slender Gram-positive rods containing endospores, with occasional filamentous cell arrangements. The isolate was motile and catalase-positive, confirming its aerobic metabolic nature. In contrast to Colony No. 1, this strain exhibited α-hemolytic activity on blood agar, indicating the production of hemolysins and suggesting a higher level of extracellular enzymatic activity.

Biochemical testing showed that Colony No. 2 was capable of fermenting mannitol and xylose, while lecithinase activity was not detected. The isolate yielded a negative Voges–Proskauer reaction, did not hydrolyze starch, and did not ferment salicin. This combination of phenotypic traits, particularly the absence of starch hydrolysis, negative Voges–Proskauer reaction, and positive hemolytic activity, is characteristic of *Bacillus mesentericus* (syn. *Bacillus amyloliquefaciens* group-related species in some classifications). Therefore, based on the overall phenotypic profile and biochemical behavior, the isolate corresponding to Colony No. 2 was tentatively assigned to the species *Bacillus mesentericus*.

### 3.2. Antimicrobial Activity of the Juices Against Bacillus spp.

The results of the antibacterial activity assessment of the tested juices against *Bacillus* spp. are summarized in Table 2.

As shown in Table 2, not all examined samples exhibited pronounced antimicrobial activity against the tested strains. The observed differences indicate considerable variability in the biological activity of the juices toward *Bacillus* spp., which appears to depend on both the botanical source of the juice and the applied dilution. The antibacterial activity of the tested juices was evaluated by broth dilution to determine MIC, followed by agar subculture to determine MBC (Table 3).

The antimicrobial activity data demonstrated high reproducibility across independent experiments, with identical MIC and MBC values obtained in all three replicates (SD = 0). This high level of consistency indicates a stable and reliable antibacterial effect of the tested cabbage juices under the applied experimental conditions.

During the evaluation of antibacterial activity, Sample No. 1 demonstrated inhibitory effects against *Bacillus mesentericus* at a dilution of 1:1 ± 0.0; however, no comparable activity was observed against *Bacillus subtilis*, indicating a strain-specific response.

In contrast, Sample No. 2 did not exhibit detectable activity against *Bacillus mesentericus*; nevertheless, a bacteriostatic effect against *Bacillus subtilis* was observed within the dilution range of 1:1 ± 0.0 to 1:4 ± 0.0, suggesting moderate inhibitory potential at higher juice concentrations.

Samples No. 3 and No. 4 demonstrated antimicrobial activity against both bacterial strains, effectively inhibiting growth at dilutions ranging from 1:1 ± 0.0 to 1:3 ± 0.0. This broader spectrum of activity suggests either a higher concentration or a more favorable composition of bioactive compounds capable of suppressing both *Bacillus* species.

Sample No. 5 exhibited inhibitory activity against *Bacillus subtilis* only at a dilution of 1:1 ± 0.0, while no effect was detected against *Bacillus mesentericus*, indicating limited antibacterial efficacy.

Overall, comparative analysis of the results indicates that Samples No. 3 and No. 4 were the most effective against *Bacillus* spp., exhibiting pronounced bacteriostatic activity characterized by the suppression of bacterial growth across multiple dilution levels.

As shown in Table 4, the antimicrobial activity observed in the present study is consistent with previously published data on *Brassica* spp. extracts. Comparable inhibitory effects against *Bacillus* spp. have been reported for cabbage juices and solvent extracts, with antimicrobial activity primarily attributed to glucosinolates, their degradation products (isothiocyanates), and polyphenolic compounds. The agreement between the present findings and literature data supports the potential of cabbage-derived phytocomponents as natural antimicrobial agents for controlling spoilage microorganisms in bread and other cereal-based products [31,56,57,58,59].

### 3.3. Effect of Plant Juices on Yeast Cells

Although the biologically active preparation demonstrated inhibitory activity against *Bacillus* spp., it is essential that it does not adversely affect *Saccharomyces cerevisiae*, which plays a key role in dough fermentation and is critical for maintaining leavening efficiency in the baking industry. To address this requirement, the effect of the investigated plant juices on the growth and metabolic activity of *S. cerevisiae* was systematically evaluated, and the results are presented in Table 5.

The experimental data indicated that antifungal activity varied among the tested juices. Samples No. 1, No. 4, and No. 5 exhibited only weak signs of inhibition; however, no clearly defined zones of growth suppression were observed. This suggests that these juices exerted minimal or sub-inhibitory effects on yeast cells and did not significantly interfere with yeast viability.

In contrast, Samples No. 2 and No. 3, obtained from napa cabbage (*Brassica rapa* subsp. *pekinensis*) and white cabbage (*Brassica oleracea* var. *capitata*), respectively, showed no detectable influence on yeast growth or metabolic activity. Yeast cells retained normal growth dynamics and metabolic performance, indicating that these juices did not exert inhibitory or stress-inducing effects. These results clearly confirm the compatibility of the tested juices with yeast-driven fermentation processes.

The observed selectivity is particularly advantageous for bakery applications, as it enables effective inhibition of spoilage and pathogenic bacteria while preserving yeast functionality. Maintaining yeast viability and metabolic activity is essential for stable dough fermentation, gas production, and the formation of desirable textural and sensory characteristics in bakery products. Therefore, selective antimicrobial action represents a critical requirement for the development of natural bio-preservatives that ensure microbiological safety without compromising technological performance or product consistency.

The obtained results are consistent with previous reports demonstrating that certain cabbage-derived juices, despite being rich in lipophilic compounds, terpenes, and sulfur-containing metabolites, are capable of selectively inhibiting pathogenic or spoilage bacteria while remaining compatible with beneficial microorganisms such as *Saccharomyces cerevisiae* [60,61,62]. The absence of inhibitory effects on yeast suggests that these juices do not interfere with key fermentative pathways, including carbohydrate metabolism, enzymatic activity involved in sugar utilization, and carbon dioxide production, all of which are crucial for effective dough leavening and structural development.

Overall, the findings indicate that cabbage-derived plant juices can serve as promising natural antimicrobial agents for bakery applications. Their ability to provide targeted inhibition of undesirable bacteria while maintaining the functional activity of yeast offers a dual advantage, contributing simultaneously to enhanced food safety and the preservation of high product quality.

### 3.4. Spectral Analysis

Natural plant extracts represent complex multicomponent systems, and complete identification of individual constituents cannot be achieved using a single analytical technique. Therefore, Fourier-transform infrared (FTIR) spectroscopy was applied as a rapid and informative tool for the preliminary characterization of the functional groups present in cabbage-derived extracts.

**Figure 2:**
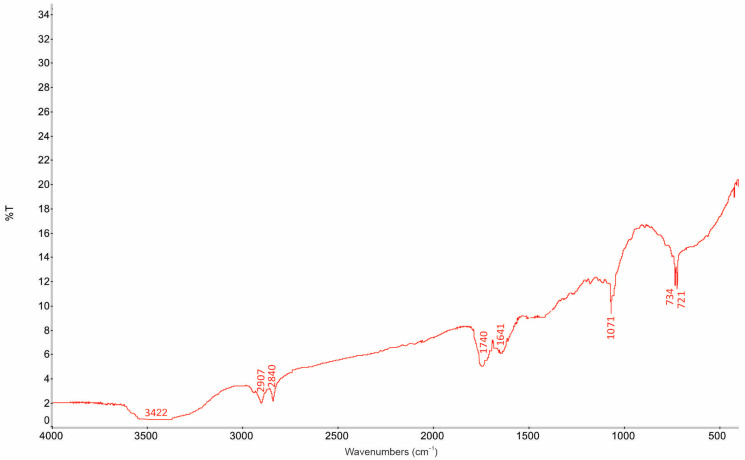
Spectral profile of the formulation obtained from the Napa cabbage extract—for Napa cabbage (Experimentally detected absorption bands (in cm^−1^): 3422 (vs, vbr), 2907 (vs), 2840 (vs), 1740 (vs, br), 1641 (vs, br), 1071 (s), 734 (vs) and 721 (vs, vsh). Legend: band intensity is indicated as s (strong), vs (very strong), and w (weak), while band width is denoted as br (broad) and vbr (very broad); peak shape is defined as sh (sharp) and vsh (very sharp) (*n* = 3).

The FTIR spectra of extracts obtained from six cabbage varieties are presented in Figure 2 and summarized in Table 6. The spectra of all samples exhibited characteristic absorption bands corresponding to aliphatic C–H stretching vibrations (2920–2850 cm^−1^), indicating the prevalence of lipid and alkane structures. Strong absorption bands observed in the carbonyl region (1740–1710 cm^−1^) confirm the presence of esterified fatty acids and other carbonyl-containing compounds, which are known to disrupt bacterial cell membranes and interfere with metabolic processes.

Since diethyl ether, a low-polarity solvent, was used for extraction, the biologically active fractions are expected to be enriched predominantly with lipophilic compounds, including fatty acids and their esters, terpenes, phytosterols, and sulfur-containing metabolites. The detection of absorption bands attributable to sulfur-containing functional groups (720–740 cm^−1^) suggests the presence of isothiocyanates and related glucosinolate degradation products, which are widely reported to exhibit antimicrobial activity against Gram-positive bacteria, including *Bacillus* spp.

Weak absorption bands observed in the hydroxyl (O–H) stretching region (~3400 cm^−1^) suggest the presence of small amounts of polar constituents, including phenolic compounds. However, it should be noted that FTIR spectroscopy provides primarily qualitative information and does not allow for the accurate quantification of individual phytochemical classes such as phenolics or flavonoids. Therefore, to quantitatively substantiate the phytochemical composition inferred from the spectral analysis, the total phenolic content (TPC) and total flavonoid content (TFC) of the selected cabbage extract were determined using established spectrophotometric assays, as described in the following section.

Overall, the FTIR analysis indicates that the biologically active fractions of the cabbage extract are dominated by lipophilic compounds with potential antibacterial activity. The combined presence of fatty acid derivatives, terpenoid structures, sulfur-containing metabolites, and minor phenolic constituents likely contributes to the observed inhibitory effects against *Bacillus* spp., acting through membrane disruption and metabolic interference mechanisms.

Table 7 presents a comparative analysis of the TPC and TFC values obtained for the napa cabbage extract investigated in this study and those reported for other *Brassica* vegetables. The TPC and TFC levels of napa cabbage were comparable to those reported for white cabbage and broccoli, while being lower than the values typically observed for red cabbage, which is characterized by a higher accumulation of phenolic compounds, particularly anthocyanins. These results confirm that napa cabbage represents a substantial source of phenolic and flavonoid compounds and provide quantitative support for the phytochemical profile suggested by FTIR spectroscopy. Variations among *Brassica* species can be attributed to genetic differences, extraction protocols, solvent polarity, and processing conditions employed in the respective studies [1,63,64].

## 4. Technical Challenges and Study Limitations

Despite the scientific relevance and promising outcomes of the present study, several technical challenges and limitations must be acknowledged when interpreting the results and considering their industrial applicability.

One of the major challenges is the intrinsic variability of plant-derived raw materials. The biochemical composition of cabbage—including lysozyme-related proteins, glucosinolates, isothiocyanates, and phenolic compounds—is strongly influenced by cultivar selection, agroclimatic conditions, maturity at harvest, and post-harvest handling. This natural variability may result in fluctuations in antimicrobial efficacy and complicates the standardization of functional performance in bakery applications. To address this issue, future studies should focus on raw material profiling and the establishment of quality criteria for bioactive-rich cabbage fractions.

Another important limitation concerns the stability of bioactive compounds during technological processing. Dough mixing, fermentation, and baking subject the system to mechanical shear, enzymatic activity, and elevated temperatures, which may partially degrade or inactivate thermolabile components. As a result, the antimicrobial and antioxidant effectiveness observed under laboratory conditions may be reduced during actual bread production. Optimization of processing parameters—such as fermentation time, temperature, and baking regime—is therefore essential to maximize the retention of functional compounds while maintaining desirable sensory and technological properties.

Furthermore, complex interactions between plant-derived bioactive components and the dough matrix constituents (gluten proteins, starch, and dietary fiber) can significantly affect dough rheology, gas-holding capacity, and crumb structure. Excessive incorporation of cabbage-based ingredients may interfere with gluten network formation, potentially leading to reduced loaf volume, increased crumb firmness, or textural defects. Consequently, precise control of dosage levels and formulation balance is critical to achieve an optimal compromise between antimicrobial functionality and bread quality.

Finally, the microbiological evaluation in this study was primarily conducted under controlled laboratory conditions. While such conditions are suitable for comparative screening and mechanistic assessment, they do not fully reflect the complexity of industrial bakery environments, where additional contamination risks may arise during cooling, slicing, packaging, and storage. Therefore, the practical effectiveness of the proposed approach should be further validated through pilot-scale trials and industrial storage studies to confirm its robustness under real production conditions.

Overall, these limitations do not diminish the scientific value of the present work but rather define clear directions for future research aimed at improving process robustness, ensuring product consistency, and facilitating industrial implementation of cabbage-derived natural antimicrobial agents in bakery products.

## 5. Future Research Perspectives

Future research should prioritize the standardization and optimization of extraction, stabilization, and characterization protocols for plant-derived bioactive compounds to ensure reproducible quality and functional performance. The application of advanced analytical techniques, including high-performance liquid chromatography (HPLC), liquid chromatography–mass spectrometry (LC–MS), and Fourier-transform infrared spectroscopy (FTIR), would enable detailed identification and quantitative profiling of key antimicrobial constituents responsible for the observed inhibitory effects.

Further investigations are warranted to assess synergistic interactions between cabbage-derived lysozyme-containing complexes and other natural antimicrobial systems, such as organic acids, bacteriocins, probiotics, or fermentation-derived metabolites. Such multi-hurdle approaches could significantly enhance microbiological stability while preserving desirable sensory and technological attributes of bakery products.

An important direction for future work involves long-term shelf-life and challenge studies conducted under realistic industrial storage and distribution conditions. These studies are essential to evaluate microbial safety, oxidative stability, and quality retention throughout the entire product lifecycle. In parallel, consumer acceptance and sensory preference studies, combined with market-oriented perception analyses, will be crucial for assessing the commercial viability and positioning of functional bread formulations enriched with natural antimicrobial agents.

Another promising avenue of research is the development of encapsulation and nano-delivery systems designed to protect bioactive compounds from thermal and mechanical degradation during processing. Encapsulation technologies may allow controlled release, improved bioavailability, and enhanced functional efficacy of antimicrobial compounds while maintaining a clean-label profile. These approaches are fully aligned with current trends in sustainable food design and could support the creation of next-generation functional bakery products characterized by improved safety, nutritional value, and extended shelf stability.

Collectively, these future research directions provide a solid scientific and technological framework for advancing plant-based antimicrobial strategies from laboratory-scale validation toward industrial implementation and commercial success.

## 6. Conclusions

During the initial screening stage, the antibacterial activity of freshly pressed juices obtained from different cabbage varieties was evaluated against thermophilic *Bacillus* spp. The results demonstrated clear differences in antimicrobial efficacy among the tested samples. Specifically, white cabbage juice effectively inhibited the growth of *Bacillus subtilis* up to a dilution of 1:4, whereas napa (Chinese) cabbage and broccoli juices exhibited pronounced antimicrobial activity against both *Bacillus* strains up to a dilution of 1:3.

In parallel, the effects of the cabbage juices on yeast cells were assessed to determine their compatibility with fermentation processes. Notably, napa cabbage juice showed no inhibitory effect on the growth or metabolic activity of *Saccharomyces cerevisiae*, indicating its technological suitability for bakery applications. In comparison with broccoli juice, napa cabbage juice demonstrated a more favorable balance between antibacterial efficacy and selectivity toward yeast cells.

Based on the combined evaluation of antimicrobial activity and fermentation compatibility, napa cabbage was identified as the most promising candidate for further investigation. Consequently, an extract was prepared from napa cabbage juice and subjected to FTIR spectroscopic analysis. The spectral characterization revealed the presence of lipophilic and sulfur-containing bioactive compounds, which may play a key role in the observed antibacterial effects against *Bacillus* spp.

Overall, these findings highlight the potential of napa cabbage-derived bioactive fractions as selective natural antimicrobial agents, supporting their further phytochemical characterization and application-oriented studies aimed at improving the microbiological safety of bakery products.

## Figures and Tables

**Table 1:** Main biochemical, morphological and staining characteristics of the isolated strains (*n* = 3).

Tests	Colony No. 1	Colony No. 2
Cell morphology and staining	Gram-positive short rods with centrally located endospores	Gram-positive slender rods forming endospores
Colony appearance	Dry, rough, light-yellow colonies	Yellowish-brown, dry, wrinkled colonies
Motility	+	+
Lecithinase activity	−	−
Catalase activity	+	+
Voges–Proskauer test	+	−
Hemolytic activity	− (no hemolysis)	+ (α-hemolysis)
Starch hydrolysis	+	−
Mannitol hydrolysis	−	+
Xylose fermentation	−	+
Salicin utilization	−	−

**Table 2:** Results of the antimicrobial activity of the samples determined by the serial dilution method (*n* = 3).

Test Sample	Test Strain	
	* Bacillus * * subtilis *	* Bacillus mesentericus *
	Minimum Bactericidal Dilution (MBD)	
sample № 1	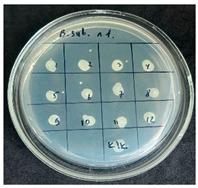	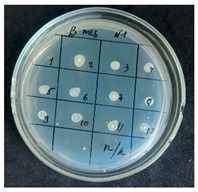
sample № 2	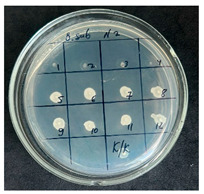	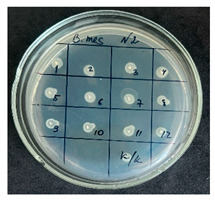
sample № 3	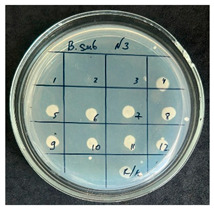	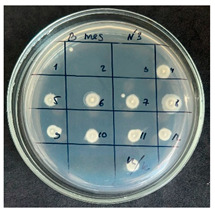
sample № 4	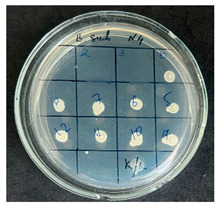	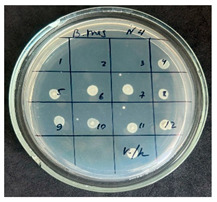
sample № 5	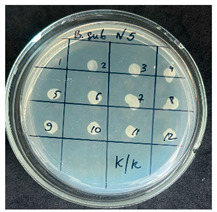	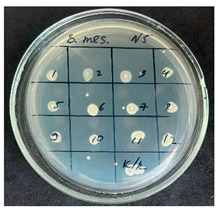

**Table 3:** MIC and MBC values (mean dilution ± SD) of cabbage juices against *Bacillus* spp. (*n* = 3).

Sample	*B. subtilis* MIC	*B. subtilis* MBC	*B. mesentericus* MIC	*B. mesentericus* MBC
1	NA	NA	1:1	NA
2	1:4 ± 0.0	1:3 ± 0.0	NA	NA
3	1:3 ± 0.0	1:3 ± 0.0	1:3 ± 0.0	1:3 ± 0.0
4	1:3 ± 0.0	1:3 ± 0.0	1:3 ± 0.0	1:3 ± 0.0
5	1:1 ± 0.0	NA	NA	NA

Note: MIC—minimum inhibitory concentration, defined as the lowest dilution showing no visible growth in broth. MBC—minimum bactericidal concentration, defined as the lowest dilution showing no growth on agar subculture. NA—no antimicrobial activity detected. All experiments were performed in triplicate (*n* = 3).

**Table 4:** Comparison of the antimicrobial activity of cabbage extracts against *Bacillus* spp. with literature data.

Study	Plant Material/Extract	Target Microorganism	Method	Reference
This study	*Brassica oleracea* juice	*Bacillus subtilis*, *Bacillus mesentericus*	Agar diffusion	Present study
Brandi et al.	*Brassica oleracea* leaf juice	*Bacillus cereus*, *E. coli*, *Salmonella Enteritidis*	In vitro growth inhibition	Brandi et al., 2006 [56]
Prasad et al.	*Brassica oleracea* ethanolic extract	*Bacillus subtilis*	Agar well diffusion	Prasad et al., 2015 [57]
Hong & Kim	*Brassica rapa* (isothiocyanates)	*Bacillus cereus*, *Staphylococcus aureus*	MIC determination	Hong & Kim, 2013 [58]
Jaiswal et al.	*Brassica oleracea* polyphenol extract	Foodborne bacteria incl. *Bacillus* spp.	Antioxidant & antibacterial assays	Jaiswal et al., 2011 [31]

**Table 5:** Results of antifungal activity of the samples determined by the serial dilution method (*n* = 3).

Test Sample	Test Strain	Test Sample	Test Strain
Sample № 1	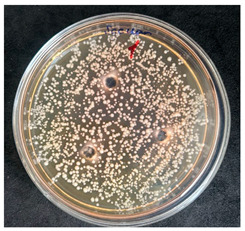	Sample № 4	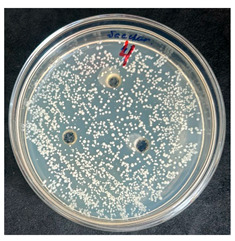
Sample № 2	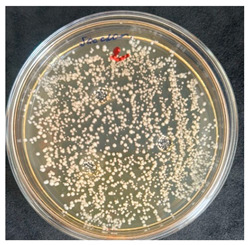	Sample № 5	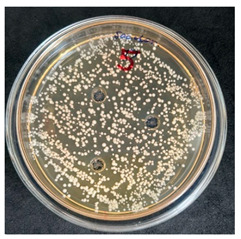
Sample № 3	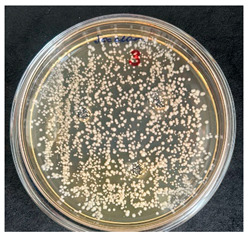		

**Table 6:** Spectral absorption bands of the bioactive fraction and their tentative assignments (*n* = 3).

Wavenumber (cm^−1^)					
**Band Range (Experimental) (cm^−1^)**	**Band Range (Literature) (cm^−1^)**	**Band No.**	**Band Interaction**	**Band Assignments**	**Possible Compounds**
3200–3600	3422	4	bend	–OH	stretching (alcohols, phenols)
2830–2960	2907	1	stretch	–CH_2_, –CH_3_	(aliphatic C–H stretching)
1735–1750	1740	2	stretch	C=O	(ester carbonyl, lipid esters)
1620–1650	1641	3	bend	C=C	stretching (aromatic or alkene)
1050–1300	1071	5	stretch	C–O/C–O–C	stretching (esters, ethers)
720–1050	734	6	stretch	S=O N–O	(sulfoxides), SO_2_ symmetric/asymmetric

**Table 7:** Comparison of total phenolic content (TPC) and total flavonoid content (TFC) of cabbage extracts with literature data.

Sample	TPC (mg GAE/g Extract)	TFC (mg QE/g Extract)	Reference
Napa cabbage (this study)	48.6 ± 2.3	21.4 ± 1.1	This study
White cabbage	32–45	12–20	Jaswal et al., 2012 [63]
Red cabbage	60–75	25–38	Podsędek, 2007 [1]
Broccoli	28–40	10–18	Vallejo et al., 2002 [64]

Values are expressed as mean ± SD (*n* = 3).

## Data Availability

The original contributions presented in the study are included in the article, further inquiries can be directed to the corresponding author.

## References

[1] Podsędek A. (2007). Natural antioxidants and antioxidant capacity of Brassica vegetables: A review. LWT-Food Sci. Technol..

[2] Bhandari S., Jo J., Lee J. (2015). Comparison of Glucosinolate Profiles in Different Tissues of Nine Brassica Crops. Molecules.

[3] Bhandari S., Kwak J.-H. (2015). Chemical Composition and Antioxidant Activity in Different Tissues of Brassica Vegetables. Molecules.

[4] Singh J., Upadhyay A.K., Prasad K., Bahadur A., Rai M. (2007). Variability of carotenes, vitamin C, E and phenolics in Brassica vegetables. J. Food Compos. Anal..

[5] Li X., Pang W., Piao Z. (2017). Omics Meets Phytonutrients in Vegetable Brassicas: For Nutritional Quality Breeding. Hortic. Plant J..

[6] Le T.N., Chiu C.-H., Hsieh P.-C. (2020). Bioactive Compounds and Bioactivities of *Brassica oleracea* L. var. Italica Sprouts and Microgreens: An Updated Overview from a Nutraceutical Perspective. Plants.

[7] Singh J., Upadhyay A.K., Bahadur A., Singh B., Singh K.P., Rai M. (2006). Antioxidant phytochemicals in cabbage (*Brassica oleracea* L. var. capitata). Sci. Hortic..

[8] Jaiswal A.K., Rajauria G., Abu-Ghannam N., Gupta S. (2011). Phenolic Composition, Antioxidant Capacity and Antibacterial Activity of Selected Irish Brassica Vegetables. Nat. Prod. Commun..

[9] Iztayev A., Kulazhanov T., Maemerov M., Iztayev B., Mamayeva L. (2018). The efficiency of ionocavitational processing and storage in the nitrogen medium of oilseeds. Acta Tech..

[10] Iztayev A., Yakiyayeva M., Kulazhanov T., Kizatova M., Maemerov M., Stankevych G., Toxanbayeva B., Chakanova Z. (2018). Efficient mathematical models of ion-ozon cavitation treatment for long-term storage of grain legume crops. Acta Tech. CSAV (Ceskoslovensk Akad. Ved).

[11] Aufartová J., Brabcová I., Torres-Padrón M.E., Solich P., Sosa-Ferrera Z., Santana-Rodríguez J.J. (2017). Determination of fluoroquinolones in fishes using microwave-assisted extraction combined with ultra-high performance liquid chromatography and fluorescence detection. J. Food Compos. Anal..

[12] Nurgozhina Z., Shansharova D., Umirzakova G., Maliktayeva P., Yakiyayeva M. (2022). The influence of grain mixtures on the quality and nutritional value of bread. Potravin. Slovak J. Food Sci..

[13] Fahey J.W., Zalcmann A.T., Talalay P. (2001). The chemical diversity and distribution of glucosinolates and isothiocyanates among plants. Phytochemistry.

[14] Rokayya S., Li C.-J., Zhao Y., Li Y., Sun C.-H. (2013). Cabbage (*Brassica oleracea* L. var. capitata) Phytochemicals with Antioxidant and Anti-inflammatory Potential. Asian Pac. J. Cancer Prev..

[15] Vallejo F., Tomás-Barberán F.A., García-Viguera C. (2002). Glucosinolates and vitamin C content in edible parts of broccoli florets after domestic cooking. Eur. Food Res. Technol..

[16] Liu R., Pei Z., Liu D., Zhao X., Mao J., Wang Y., Hu J., Zhou P. (2025). Natural inhibitor combinations targeting α-amylase and α-Glucosidase: A food-derived strategy for safer type 2 diabetes management. Food Biosci..

[17] Arrais A., Testori F., Calligari R., Gianotti V., Roncoli M., Caramaschi A., Todeschini V., Massa N., Bona E. (2022). Extracts from Cabbage Leaves: Preliminary Results towards a “Universal” Highly-Performant Antibacterial and Antifungal Natural Mixture. Biology.

[18] Favela-González K.M., Hernández-Almanza A.Y., De la Fuente-Salcido N.M. (2020). The value of bioactive compounds of cruciferous vegetables (Brassica) as antimicrobials and antioxidants: A review. J. Food Biochem..

[19] Ferraboschi P., Ciceri S., Grisenti P. (2021). Applications of Lysozyme, an Innate Immune Defense Factor, as an Alternative Antibiotic. Antibiotics.

[20] Sibi G., Shukla A., Dhananjaya K.R., Ravikumar K.R., Mallesha H. (2013). In vitro antibacterial activities of Broccoli (*Brassica oleracea* L. var. italica) against food borne bacteria. J. Appl. Pharm. Sci..

[21] Rahmani M., Esmaeili A., Taherkhani M. (2024). Antimicrobial Potential of *Brassica oleracea* Extracts (White and Broccoli) and Their Resistance Compared to Doxycycline Against Gram-Positive and Gram-Negative Bacteria. Food Sci. Nutr..

[22] Vale A.P., Santos J., Melia N., Peixoto V., Brito N.V., Oliveira M.B.P.P. (2015). Phytochemical composition and antimicrobial properties of four varieties of *Brassica oleracea* sprouts. Food Control.

[23] Waghulde S., Khan N.A., Gorde N., Kale M., Naik P., Yewale R.P. (2019). Comparative Antimicrobial Activity Study of *Brassica oleceracea*. Proceedings.

[24] Mejías N., Vega-Galvez A., Gomez-Perez L.S., Pasten A., Uribe E., Cortés A., Valenzuela-Barra G., Camus J., Delporte C., Bernal G. (2024). Health-Promoting Properties of Processed Red Cabbage (*Brassica oleracea* var. capitata f. *rubra*): Effects of Drying Methods on Bio-Compound Retention. Foods.

[25] Iztayev A., Urazaliev R., Yakiyayeva M., Maemerov M., Shaimerdenova D., Iztayev B., Toxanbayeva B., Dauletkeldі Y. (2018). The Investigation of The Impact of Dynamic Deterioration of Ozone on Grass Growth and the Consequence of Ion-Ozone Cavitation Treatment. J. Adv. Res. Dyn. Control Syst..

[26] Kavishri S., Panneer Selvam S., Shanmugam R., Ramadoss R., Sundar S., Ramani P. (2024). Exploring the Antimicrobial Potential and Cytotoxic Effects of Different Brassica oleracea Varieties. Cureus.

[27] Schafhauser T., Kulik A. (2022). Isolation and Purification of Natural Products from Microbial Cultures. Methods in Molecular Biology.

[28] Dal Prá V., Dolwitsch C.B., Lima F.O., de Carvalho C.A., Viana C., do Nascimento P.C., da Rosa M.B. (2015). Ultrasound-Assisted Extraction and Biological Activities of Extracts of *Brassica oleracea* var. capitata. Food Technol. Biotechnol..

[29] Alani H., Ozaslan M., Karagoz I.D., Simitcioglu B. (2021). Investigation of Antimicrobial, DNA Protective and Cytotoxic Activity of Red Cabbage (*Brassica oleracea* L. Var. *Capitata* F. *rubra*) Plant. Eurasia Proc. Sci. Technol. Eng. Math..

[30] Jabeen A., Mir J.I., Malik G., Yasmeen S., Ganie S.A., Rasool R., Hakeem K.R. (2024). Biotechnological interventions of improvement in cabbage (*Brassica oleracea* var. *capitata* L.). Sci. Hortic..

[31] Jaiswal A.K., Abu-Ghannam N., Gupta S. (2011). A comparative study on the polyphenolic content, antibacterial activity and antioxidant capacity of different solvent extracts of *Brassica oleracea* vegetables. Int. J. Food Sci. Technol..

[32] Egea M.B., Fernandes S.S., Braga A.R.C., Lemes A.C., de Oliveira Filho J.G. (2025). Bioactive Phytochemicals from Red Cabbage (*Brassica oleracea*) By-products. Bioactive Phytochemicals in By-Products from Leaf, Stem, Root and Tuber Vegetables.

[33] Hu S., Wang J., Kung H., Wang J., Lee W., Yang Y. (2004). Antimicrobial Effect of Extracts of Cruciferous Vegetables. Kaohsiung J. Med. Sci..

[34] Upadhyay R., Sehwag S., Singh S.P. (2015). Antioxidant Activity and Polyphenol Content of *Brassica oleracea* Varieties. Int. J. Veg. Sci..

[35] Mokaizh A.A.B., Nour A.H., Alazaiza M.Y.D., Mustafa S.E., Omer M.S., Nassani D.E. (2024). Extraction and Characterization of Biological Phytoconstituents of *Commiphora gileadensis* Leaves Using Soxhlet Method. Processes.

[36] Jeon K.-H., Heo S.-H., Lee Y.-L., Hwang E.-J., Hyun J.-Y., Lee Y.-J., Park J.-W., Yi H.-Y., Chun J.-Y. (2024). Antioxidant Activity of Broccoli and Cabbage Depending on the Extraction Solvent. Food Eng. Prog..

[37] Araújo A.C.d., Gomes J.P., Silva F.B.d., Nunes J.S., Santos F.S.d., Silva W.P.d., Ferreira J.P.d.L., Queiroz A.J.d.M., Figueirêdo R.M.F.d., Lima G.S.d. (2023). Optimization of Extraction Method of Anthocyanins from Red Cabbage. Molecules.

[38] Xu H., Fu L., Li J., Lin X., Chen L., Zhong F., Hou M. (2024). A Method for Analyzing the Phenotypes of Nonheading Chinese Cabbage Leaves Based on Deep Learning and OpenCV Phenotype Extraction. Agronomy.

[39] Yiğit Ü., Turabi Yolaçaner E. (2024). Enhanced extraction of phenolic compounds from red cabbage utilizing microwave-assisted method: A Box-Behnken approach for optimization. Food Mater. Res..

[40] Wan J., Bao H., Huang L., Ding Y., Chen Z., Zhu C. (2019). Soil cadmium extraction in Chinese cabbage and cabbage intercropping. Ciência Rural.

[41] Meng L., Ding P., Tan Y., Zhang Y., Zhao J. (2025). Study on the Ultrasonic-Assisted Extraction Process of Anthocyanin from Purple Cabbage with Deep Eutectic Solvent. Molecules.

[42] Iztayev B., Iztayev A., Kulazhanov T., Iskakova G., Yakiyayeva M., Muldabekova B., Baiysbayeva M., Tursunbayeva S. (2024). A Study of the Influence of Ion-Ozonized Water on the Properties of Pasta Dough Made from Wheat Flour and Pumpkin Powder. Foods.

[43] Tungyshbayeva U., Mannino S., Uazhanova R., Adilbekov M.A., Yakiyayeva M.A., Kazhymurat A. (2021). Development of a methodology for determining the critical limits of the critical control points of the production of bakery products in the Republic of Kazakhstan. East.-Eur. J. Enterp. Technol..

[44] Neslihan G., Bahar G., Pinar I., Huseyin E. (2024). Determination of antimicrobial activities and DNA protection properties of Scytosiphon lomentaria extracts. GSC Biol. Pharm. Sci..

[45] Pin J., Guo T., Xv M., Zou X., Hu W. (2025). Fast extraction of navigation line and crop position based on LiDAR for cabbage crops. Artif. Intell. Agric..

[46] Harrigan W.F., McCance M.E. (1998). Laboratory Methods in Food Microbiology.

[47] Jay J.M., Loessner M.J., Golden D.A. (2005). Modern Food Microbiology.

[48] Doyle M.P., Buchanan R.L. (2012). Food Microbiology.

[49] Bishnoi A., Jyani M., Bhatia S. (2026). Valorization of Sweet Lime (Mosambi) Peel and Pomace for Pectin Extraction: Process Optimization and Functional Characterization. Int. J. Adv. Res. Sci. Commun. Technol..

[50] André S., Vallaeys T., Planchon S. (2017). Spore-forming bacteria responsible for food spoilage. Res. Microbiol..

[51] Snyder A.B., Martin N., Wiedmann M. (2024). Microbial food spoilage: Impact, causative agents and control strategies. Nat. Rev. Microbiol..

[52] Trujillo M.E., Dedysh S., DeVos P., Hedlund B., Kämpfer P., Rainey F.A., Whitman W.B. (2015). Bergey’s Manual of Systematics of Archaea and Bacteria.

[53] Brenner D.J., Staley J.T., Krieg N.R. (2005). Classification of Procaryotic Organisms and the Concept of Bacterial Speciation. Bergey’s Manual^®^ of Systematic Bacteriology.

[54] Zhakatayeva A., Iztayev А., Мuldabekova B., Yakiyayeva М., Hrivna L. (2020). Scientific security assessment of safety risk of raw sugar products. Periódico Tchê Química.

[55] Cappuccino J.G., Sherman N. (2002). Microbiology: A Laboratory Manual.

[56] Brandi G., Amagliani G., Schiavano G.F., De Santi M., Sisti M. (2006). Antibacterial activity of *Brassica oleracea* leaf juice against foodborne pathogens. J. Food Prot..

[57] Prasad M.R., Srinivasan R., Kumar S., Ramesh B. (2015). In vitro antimicrobial activity of selected Brassicaceae plant extracts against foodborne pathogens. J. Appl. Pharm. Sci..

[58] Hong E., Kim G.H. (2013). Antimicrobial and anti-inflammatory activities of β-phenylethyl isothiocyanate from *Brassica rapa*. J. Food Sci..

[59] Somasundaram J., Moni S.S., Makeen H.A., Intakhabalam M., Pancholi S.S., Siddiqui R., Eltyepelmobark M. (2018). Antibacterial potential of ethanolic extract of broccoli (*Brassica oleracea* var. *italica*) against human pathogenic bacteria. Int. J. Pharm. Res..

[60] Protska V., Fedosov A., Burda N., Dobrovolnyi O., Dababneh M.F., Kuznetsova M., Zhuravel I., Budanova L. (2021). The study of volatile fractions of cabbage leaves (*Brassica oleracea* L. convar. capitata (L.) Alef. var. alba DC.) and determination of its antibacterial and antifungal activity. Thai J. Pharm. Sci..

[61] Demirdöven A., Özdoğan K., Erdoğan-Tokatlı K. (2015). Extraction of Anthocyanins from Red Cabbage by Ultrasonic and Conventional Methods: Optimization and Evaluation. J. Food Biochem..

[62] Rubab M., Chelliah R., Saravanakumar K., Kim J.-R., Yoo D., Wang M.-H., Oh D.-H. (2020). Phytochemical characterization, and antioxidant and antimicrobial activities of white cabbage extract on the quality and shelf life of raw beef during refrigerated storage. RSC Adv..

[63] Jaswal A., Abu-Ghannam N., Gupta S. (2012). Effect of extraction conditions on antioxidant activity, total phenolic content and antibacterial activity of cabbage extracts. Food Chem..

[64] Vallejo F., Tomás-Barberán F.A., García-Viguera C. (2002). Phenolic compound content of fresh and processed broccoli. J. Sci. Food Agric..

